# A Study of Speech Recognition for Kazakh Based on Unsupervised Pre-Training

**DOI:** 10.3390/s23020870

**Published:** 2023-01-12

**Authors:** Weijing Meng, Nurmemet Yolwas

**Affiliations:** 1 Xinjiang Multilingual Information Technology Laboratory, Urumqi 830017, China; 2 College of Information Science and Engineering, Xinjiang University, Urumqi 830017, China

**Keywords:** automatic speech recognition, Factorized TDNN, unsupervised pre-training, speech synthesis

## Abstract

Building a good speech recognition system usually requires a lot of pairing data, which poses a big challenge for low-resource languages, such as Kazakh. In recent years, unsupervised pre-training has achieved good performance in low-resource speech recognition, but it is rarely used in Kazakh and other Central and West Asian languages. In this paper, wav2vec2.0 is improved by integrating a Factorized TDNN layer to better preserve the relationship between the voice and the time step before and after the quantization, which is called wav2vec-F. The unsupervised pre-training strategy was used to learn the potential speech representation from a large number of unlabeled audio data and was applied to the cross-language ASR task, which was optimized using the noise contrast binary classification task. At the same time, speech synthesis is used to promote the performance of speech recognition. The experiment shows that wav2vec-F can effectively utilize the unlabeled data from non-target languages, and the multi-language pre-training is obviously better than the single-language pre-training. The data enhancement method using speech synthesis can bring huge benefits. Compared with the baseline model, Librispeech’s test-clean dataset has an average reduction of 1.9% in the word error rate. On the Kazakh KSC test set, the pre-training using only Kazakh reduced the word error rate by 3.8%. The pre-training of a small amount of Kazakh speech data synthesized by multi-language combined with TTS achieved a word error rate of 8.6% on the KSC test set when the labeled data were only 10 h, which was comparable to the results of the previous end-to-end model when the labeled data were 30 times less.

## 1. Introduction

Compared with traditional automatic speech recognition frameworks [1], divided into acoustics, pronunciation, and language modelling, sequence-based models [2,3,4,5,6] have shown remarkable performance in speech recognition tasks over the recent years. They directly use neural networks to learn speech-to-text mapping, avoiding complex modelling processes. Transformer [7] is a widely used sequence-to-sequence model that has proven to be a fantastic tool for building end-to-end speech recognition systems [8,9,10]. However, the end-to-end approach requires a large amount of annotated data to complete the training to achieve a good performance, which poses a significant challenge [11] to some low-resource languages that cannot meet the requirements of markup data for end-to-end modelling. Unmatched data are more accessible to collect than labelled data. Therefore, it is worth exploring how to use unpaired speech and text data to improve the performance of a low-resource speech recognition system under the constraints of limited annotation data.

Two main strategies have been proposed to make the most of unmatched data: unsupervised pre-training and semi-supervised learning. In the aspect of unsupervised pre-training, the bidirectional encoder representation of Transformers (BERT) [12] and generative pre-training (GPT) [13] in the field of natural language processing use a large number of unlabeled data to conduct pre-training and learn general feature representation. The training target is only related to the acoustic features themselves. Fine-tuning can transfer the learned knowledge to the downstream task, considerably speeding up the convergence of the model. In semi-supervised learning [14,15,16], encoders are usually used to reconstruct a large number of unpaired data to enhance the feature extraction of a small number of paired data. In the field of computer vision, unsupervised pre-training also shows broad application prospects for tasks such as, capturing statistical data [17], learning bias [18], and object detection [19].

In the field of speech recognition, researchers have also proposed some methods of unsupervised pre-training. Contrastive Predictive Coding (CPC) [20] combines autoregressive modelling and noise contrastive estimation with predictive coding to extract speech representations from high-dimensional data in an unsupervised manner by predicting future information. Wav2vec [21] applies CPC to speech recognition tasks, trains on large amounts of unlabeled audio data, uses the resulting representations to improve the acoustic model, and achieves a better feature extractor than manual designs. By incorporating quantization modules into the wac2vec model to discretize continuous acoustic features into a specific dictionary, the Vq-wav2vec [22] improves on the latest level of The Wall Street Journal and TIMIT benchmarks by utilizing BERTs pre-training. Wav2vec 2.0 [23] fuses BERTs sequential masking modelling with discrete CPC methods into a model that masks speech input in potential spaces and solves quantificationally defined contrast tasks in the possible representation of co-learning, showing feasibility in low-resource speech recognition tasks. Autoregressive predictive coding (APC) [24], which learns general speech representations that can be transferred to different tasks on different data sets, aims to preserve information for a wide range of downstream tasks and does not require any speech or word boundary labels, allowing the model to benefit from large amounts of unlabeled data. Jiang D et al. [25] applied APC to speech recognition tasks and effectively reduced the size of downstream marker data and model parameters while improving the recognition effect. In addition to CPC and APC, there is an unsupervised pre-training method called masking prediction coding (MPC) [26], which uses a similar structure to that used in BERT Masked-LM(MLM) to predict the coding of Transformer-based models.

Among all the languages in the world, low-resource languages account for a large proportion [11]. However, most of the current mature speech recognition systems are based on several common languages. Although researchers have conducted some research [27,28,29] on speech recognition under low-resource conditions, the research on speech recognition systems of low-resource languages such as Kazakh and some other Central and Western Asian languages is still in the initial stage. Their lack of resources is reflected in audio, text, pronunciation dictionaries, and phoneme collections. Inspired by wav2vec 2.0 and MPC, this paper integrates Factorized TDNN layers [30] in wav2vec 2.0 to reduce the potential speech feature loss when speech passes through the quantization module. Therefore, the proposed method is called wav2vec-F. At present, the unsupervised pre-training system for Kazakh speech recognition has not been well studied. We consider the cases of single-language and multi-language pre-training and adopt the complementary approach of ASR and TTS to promote low-resource Kazakh speech recognition tasks. We evaluated wav2vec-F on Librispeech and the Kazakh dataset KSC and compared the recognition results of replacing TDNN-F with other types of network layers. The experimental results show that the combination of wav2vec 2.0 and Factorized TDNN method can better preserve the relationship between the time steps before and after speech quantization so as to retain more speech features and prove the feasibility of this model in cross-language knowledge transfer.

## 2. Related Work

In this section, we briefly review the work related to this article in three sections: BERT, CPC, and wav2vec2.0.

### 2.1. BERT

**Figure 1:**
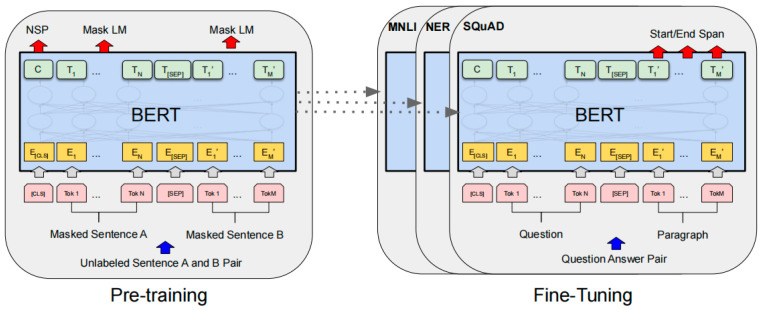
Overall pre-training and fine-tuning procedures for BERT. Apart from output layers, the same architectures are used in both pre-training and fine-tuning. The same pre-trained model parameters are used to initialize models for different down-stream tasks. During fine-tuning, all parameters are fine-tuned.

BERT is a bidirectional language representation model proposed by Jacob Devlin et al., which has two steps: pre-training and fine-tuning. The model is trained on unlabeled data on different pre-training tasks in the pre-training process. For fine-tuning, the BERT model is first initialized with pre-trained parameters, and all parameters are fine-tuned using labeled data obtained from downstream tasks. A notable feature of BERT is its unified architecture across different tasks. The difference between the pre-trained architecture and the final downstream architecture is slight. Figure 1 shows the two-stage training process of BERT.

### 2.2. CPC

**Figure 2:**
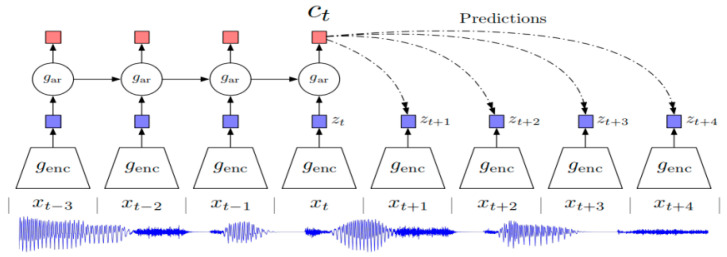
Overview of Contrastive Predictive Coding.

Contrastive Predictive Coding (CPC) is a general unsupervised learning method proposed by Aaron et al. It uses the next step prediction to learn the representation of the high-dimensional signal in an unsupervised manner. The model is mainly composed of two parts: the nonlinear encoder *g_enc_* and the autoregressive model *g_ar_*. First, given an input speech signal *x* = (*x*_1_, *x*_2_, …, *x*_T_), *g_enc_* will encode it into a potential embedding space *f_t_* = *g_enc_* (*x*_T_) with a low temporal resolution, and then *f_t_* is fed to *g_ar_* which generates a context representation *c_t_* = *g_ar_* (*z_t_*). Figure 2 shows the architecture of Contrastive Predictive Coding models.

The CPC model is optimized by minimizing noise contrast estimation (NCE)-based loss. At each time *t*, given a context representing *c_t_* and its *K* future embeddings {*f_t+__k_*}_1≤_*_k_*_≤*K*_, the loss is defined as follows:

(1) $$L_{t}=-\frac{1}{K}\sum_{k=1}^{K}\log[\frac{\exp(f_{t+k}^{T}h_{k}(c_{t}))}{\sum {}_{\tilde{f}\in N_{t}}\exp({\tilde{f}}^{T}h_{k}(c_{t}))}]$$

where *N_t_* is a set of negative embedded samples and *h_k_*(·) is the transformation of *k* at each step.

### 2.3. Wav2vec 2.0

**Figure 3:**
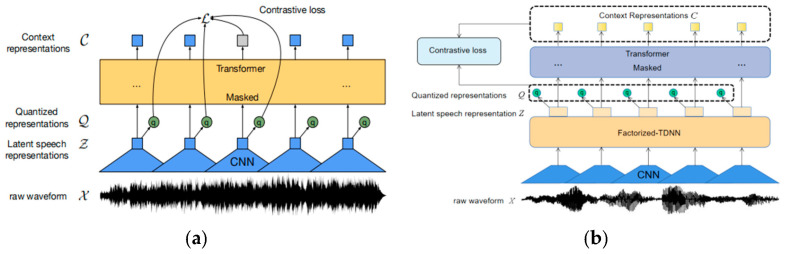
(**a**) wav2vec2.0; (**b**) wav2vec-F.

The Wav2vec2.0 model architecture is shown in Figure 3a. It is a framework for self-supervised learning from the raw audio data. The original audio is encoded by a multi-layer CNN, and then the generated latent representation is masked by a method similar to masking language modeling, which is fed to the Transformer network to generate speech representation and trained by comparison tasks.

#### 2.3.1. Quantitative Representation

**Figure 4:**
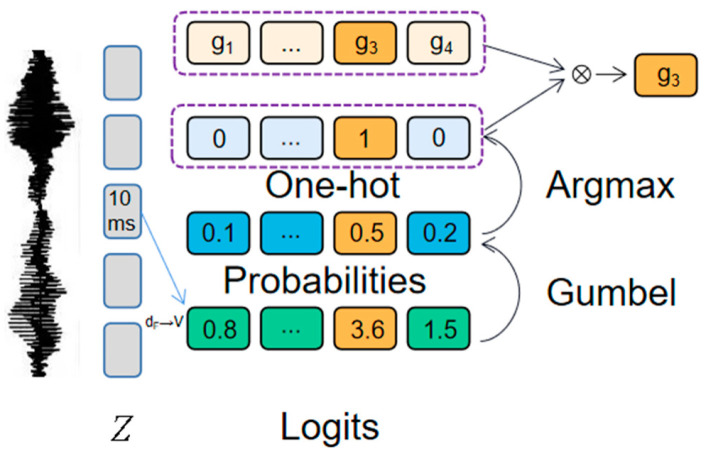
Gumbel softmax.

Learn discrete units in step one, then the context representations. Product quantization is used to discretize the output of a feature encoder into a finite set of speech representations. The role of product quantization is to select quantized representations from multiple codebooks and connect them. Given the number of codebooks *G*, each codebook contains *V* items *e* (*e* ∈ R*^V^*^×*D*/*G*^). Select an entry from each codebook and concatenate the resulting vectors *e*_1_, …, *e*_G_, then apply the linear transformation R*^d^* ↦ R*^f^* to obtain q ∈ R*^f^*. In the process of forward propagation, finding the items in the codebook corresponding to the maximum value is equivalent to a discrete operation, but this step is not derivable and cannot carry out back propagation. In order to solve this problem, the Gumbel softmax [31] method is adopted; the principle is shown in Figure 4, and the formula is:

(2) $$p_{g,v}=\frac{\exp((l_{g,v}+n_{v})/\tau )}{\sum {}_{k=1}^{V}\exp((l_{g,k}+n_{k})\tau )}$$

where *n* = −log(−log(*u*)), *u* is uniformly sampled from 0 to 1. During the forward propagation, the codeword *i* is selected by *i* = argmax*_j_ p_g_*_,*j*_, and during the reverse transmission, the true gradient of the Gumbel softmax output is used.

#### 2.3.2. Comparative Training

Context representation *C* is used for contrast learning and is conditional on masking the latent speech representation *Z*. It is necessary to identify the authentic quantified latent speech representation in a masking time step within a set of interference samples. Unlike autoregressive training, contrast training requires the model to distinguish the masking time step representations from the other time step representations. The change from the regression task to the classification task led to more effective self-training.

## 3. Methods

### 3.1. Model

The proposed model is shown in Figure 3b. It is composed of multiple convolutional neural network layers, factorized delay neural network layers, and Transformer layers.

**Figure 5:**
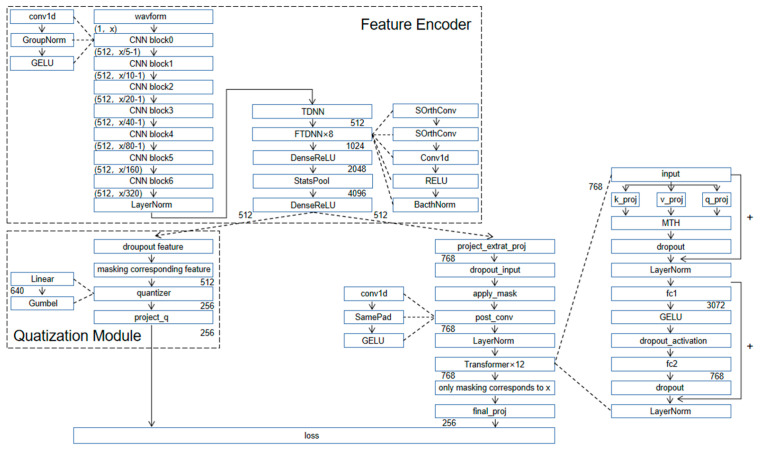
Illustration of the overall network structure of our proposed model.

The feature encoder is composed of a convolutional neural network and a factorized delay neural network. It takes the original audio *X* as an input to generate the latent speech representation *Z* = *z*_1_, …, *z*_T_ for *T* time steps. Before inputting the Transformer, *Z* is randomly sampled at a certain proportion of *p* for all time steps as the starting time step of the mask, and the mask operation is performed on the *M* time steps after that. During the mask operation, each potential speech representation *Z* of a segment of speech is regarded as a candidate starting time step with a probability *p*. The Transformer layer captures high-level content from *Z* in a similar way to [2] to produce a contextual representation of *C*. At the same time, the quantization module discretizes *Z* into a finite set of speech representations, using the method of product quantization [32] in the discretization process. The network structure of the model is shown in Figure 5.

### 3.2. Loss Function

The loss function *L* is divided into two parts, including the contrastive loss *L_m_* and the codebook diversity loss *L_d_* for the feature encoder:

(3) $$L=L_{m}+\alpha L_{d}$$

where *α* is a tuned hyperparameter.

In the pre-training process, the contextual output *c_t_* corresponding to the time step *t* of the mask is given. The model needs to select the correct quantization representation *q_t_* in a set of *K* + 1 samples $\tilde{q}$ ∈ *Q_t_* which includes *q_t_* and *K* negative samples. Negative samples are randomly and uniformly sampled at other mask time steps in the same sequence. The contrastive loss is defined as:

(4) $$L_{m}=-\log\frac{\exp(sim(c_{t},q_{t})/k)}{\sum {}_{\tilde{q}\sim Q_{t}}\exp(sim(c_{t},{\tilde{q}}_{t})/k)}$$

where we use *sim*(*c*, *q*) = *c^T^q/*‖*c*‖ · ‖*q*‖ to compute cosine similarity between context representations *c_t_* and quantized latent speech representations *q_t_*.

The contrastive task depends on the positive and negative examples of the codebook representation, while the diversity loss *L_d_* is designed to increase the use of quantization codebook $\bar{p}$*_g_* representations:

(5) $$L_{d}=\frac{1}{GV}\sum_{g=1}^{G}\sum_{V=1}^{V}{\bar{p}}_{g,v}\log{\bar{p}}_{g,v}$$

## 4. Experimental Setup

### 4.1. Datasets

In this paper, four language speech datasets of English, Chinese, Uygur, and Kazakh are used to complete all the experiments. Table 1 presents the details of these datasets.

The English speech data were obtained using the speech dataset Librispeech [33], which contains about 1000 h of speech and has been carefully segmented and aligned. This paper adopts the train-clean-100 subset, which has about 100 h of speech data, 251 speakers, and a total of 28,541 speeches. In order to compare with wav2vec 2.0, a larger 960 h of train-clean voice data are also used as unlabeled data for training to test the performance.

The Chinese speech Corpus uses Primewords Chinese Corpus Set 1, a speech dataset established by Shanghai Yuan Language Information Technology Co., Ltd. This dataset contains 100 h of Chinese speech data, with more than 98% transcription accuracy and a confidence level of 95%. There are 256 speakers and 50,384 voices in total.

The voice data from Uyghur language uses the train-clean-100 subset of the 1000 h Uyghur language voice data set in our laboratory. There are 198 speakers and 58,333 voices in total.

The Kazakh speech data set uses KSC [34], which contains about 330 h of Kazakh speech data. In this paper, speech data with different time length settings are randomly selected as fine-tuning data, and the verification and test sets of the divided standards are used. The text data used in the speech synthesis system uses the 40 h Kazakh speech data set of our laboratory, and 2000 pieces of labeled text are randomly selected for speech synthesis. About 4 h of speech data are obtained.

### 4.2. Pre-Training Configuration

The CNN layer has 7 hidden layers, each CNN layer contains a temporal convolution, layer normalization, and a GELU activation function. The temporal convolution of each block has 512 channels, the width of the convolution kernel is (10, 3, 3, 3, 3, 2, 2, 2), the stride size is (5, 2, 2, 2, 2), the stride length is about 20 ms, and the receptive field is 25 ms. Factorized-TDNN layer has 13 hidden layers, which is composed of 1 TDNN layer, 8 TDNNF layers, 3 DenseReLU layers, and 1 StatsPool layer. Each TDNN-F layer contains 2 SOrthConv layers, 1 temporal convolution, batch normalization, and a RELU function. The architecture of Factorized-TDNN is shown in Table 2.

**Figure 6:**
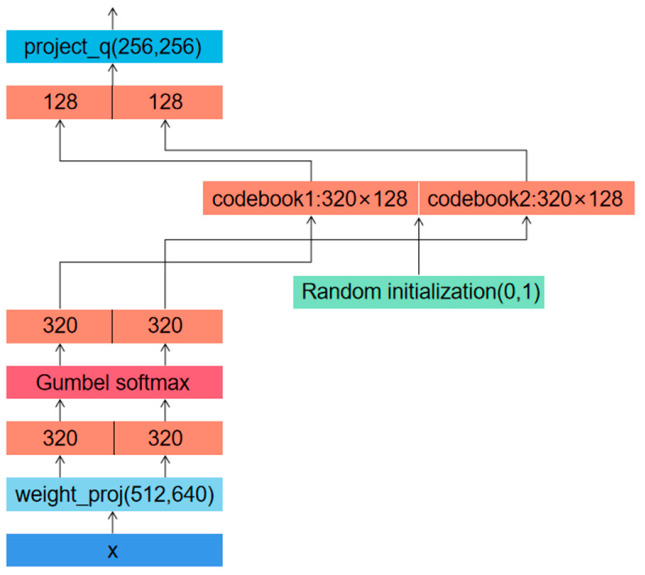
Illustrates the calculation flow inside the quantization module.

The self-attention layer consists of a 12-layer, 768-dimensional Transformer layer with eight self-attention heads. For the mask operation, *p* is chosen to be 0.065, and *M* is chosen to be 10. The quantization module gives the number of codebooks *G* = 2, the number of entries in each codebook *V* = 320, and the dimension of entries 128. The calculation process inside the quantization module is shown in Figure 6. In Equation (2), *l* is the vector of dimension (2,320), and τ controls the distribution of the sampling structure and anneals from 2 to 0.5 with a multiple of 0.999995 at each update. The learning rate is set to 5 × 10^−4^ and is optimized when using Adam [35], where the learning rate warms up in the first 10% of updates, remains constant in the next 40%, and then decays linearly in the rest. In the loss function (Equation (3)), *α* is set to 0.1. In the contrast loss function (Equation (4)), we use *k* = 0.1 and *K* = 100. The whole experiment was conducted on 1 NVIDIA GeForce RTX 3090 graphics cards with batch size set to 4, and pre-training stopped at 100 epochs.

### 4.3. Modeling Unit

Pre-training data contains English (LS), Chinese (Ma), the Uygur language (Uy), and the Kazakh (KSC). For different languages, different modeling units are selected. Chinese is a character-based writing system, so subwords are used as modeling units. The modeling units of English and Uyghur are determined by BPE algorithm [36], see Table 3 for details.

### 4.4. The TTS Configuration

Using the ESPnet-TTS toolkit [37] to create end-to-end speech synthesis system based on Tacotron 2 [38], following the configuration of LJSpeech [39]. The input of the model is a character sequence consisting of 42 Cyrillic letters and 1 symbol (“|“), the output is a set of Mel filter group characteristics of 80 d sequence. The WaveGAN [40] vocoder is used to convert these acoustic features into time-domain waveform samples without any additional speech preprocessing, such as filtering and normalization. In the Tacotron 2 system, the encoder module is modeled as a bidirectional LSTM layer with 512 units (256 units in each direction) and the decoder module is modeled as a stack of two unidirectional LSTM layers with 1024 units. The Adam algorithm was used to optimize the parameters with an initial learning rate of 10^−3^ and 200 epochs of training. To regularize the parameters, set the dropout rate to 0.5.

### 4.5. Pre-Training and Fine-Tuning

Firstly, the audio data from Librispeech 960 h is pre-trained by wav2vec 2.0 and WAV2VEC-F, respectively. After the pre-training, the pre-trained model is fine-tuned on the labeled data, and the same data set as [33] is used for fine-tuning. Next, the audio data from KSC 330 h is pre-trained with the above two models, respectively. The same data set as [34] is used for fine-tuning, and the results are compared with the previous experimental results of DNN-HMM, E2E-LSTM, and E2E-Transformer. Finally, 100 h of audio data from English, Chinese, and Uyghur were used for pre-training to obtain the monolingual model. Then, pairwise combination was used for pre-training to obtain the bilingual model. Next, 100 h audio data from each of the three languages were used for pre-training to obtain the multilingual model. A total of 2000 text data were randomly selected from the 40 h Kazakh language data set of our laboratory and synthesized into speech using the Kazakh TTS model. The multilingual model containing the target language was obtained by pre-training with the three languages at the same time. The speech data from 10 min, 1 h, 5 h, 10 h, and 20 h in the KSC training set were randomly selected as the fine-tuning data.

### 4.6. Decoding

After the model is fine-tuned, the 4-g language model is used for decoding, and Kenlm [41] is used to train the 4-g language model on the KSC LM corpus. In the decoding process, a beam search decoder [42] is used, and the beam is set to 1500.

### 4.7. Supervised Model Comparison Experiment

The DNN-HMM model was constructed using the Kaldi framework, and the “nnet3 + chain” setting was adopted according to the formula of The Wall Street Journal (WSJ). The acoustic model also adopted TDNN-F, and the meshless maximum interaction information (LF-MMI) training standard was used. The input was MFCC features. cepstral mean and variance normalization were extracted every 10 ms in a 25 ms window, and a 3-g language model based on SRILM was used for decoding.

The E2E model is constructed using the ESPnet framework and follows the formula of WSJ. The CTC criterion trains two different coding–decoder architectures based on LSTM and Transformer. The input speech is a filter bank feature of 80 dimensions, the stride length is about 10 ms, and the receptive field is 25 ms. The encoder module based on LSTM consists of three bidirectional LSTM layers, each layer has 1024 units in each direction, and the decoder module is a unidirectional LSTM with the initial learning rate set to 1. The model is trained for 20 epochs using the Adadelta optimizer. The Transformer-based system consists of 12 encoders and 6 decoders, with 4 self-attention layer heads and 256 dimensions of hidden states. The feedforward network dimension was set to 2048. The dropout rate was 0.1, and the initial learning rate was 10. A total of 160 epochs were trained with the Noam optimizer. A language model constructed from two layers of RNN with 650 LSTM units using the annotations of the training set is used for decoding.

The above three models all use speed perturbation of 0.9, 1.0, and 1.1. Meanwhile, SpecAugment is also used for data augmentation.

## 5. Results

In this paper, the wav2vec 2.0 architecture is used for pre-training as the baseline system, and unsupervised pre-training is carried out in the proposed model in different languages. By training the single-language model and multi-language model, it is proved that the proposed model can effectively learn cross-language speech representation in an unsupervised way. Moreover, the influence of language similarity on the cross-language transfer is analyzed.

### 5.1. Pre-Training for the Librispeech 960 h

The baseline and proposed model are used for training on all training subsets of Librispeech, and fine-tuning is performed on the marked 10 min, 1 h, 10 h, and 100 h data sets that are equally divided as [33]. The evaluation results on test-clean are shown in Table 4. It can be seen that WAV2VEC-F is better than the baseline model when using the same length of marked data to fine-tune, and the average word error rate is reduced by 1.9% compared with wav2vec 2.0. The audio is passed into the Factorized-TDNN layer through the convolutional neural network and is then quantized to retain more context information.

### 5.2. Pre-Training for KSC 330 h

For pre-training using only Kazakh, the training set of KSC is pre-trained as unlabeled original audio. Then, the model is fine-tuned using the validation set and evaluated using the validation and test set. Compared with the three supervised models of DNN-HMM, E2E-LSTM, and E2E-Transformer, the experimental results are shown in Table 5. Without SpeedPerturb and SpecAugment, the WER of wav2vec-F on the validation and test sets are 6.1% and 5.0%, respectively, which are 39% and 42.5% lower than that of the E2E-Transformer model, and 4.7% and 3.8% lower than that of the baseline model.

### 5.3. Pre-Training for Multiple Languages

First, the baseline model (wav2vec 2.0) and wav2vec-F are pre-trained with 100 h audio data in English, Chinese, and Uygur, respectively. The labeled Kazakh data from 10 min, 1 h, 5 h, 10 h, and 20 h are fine-tuned, and the test set of the KSC is used for evaluation. The results are shown in Table 6. It can be seen that the word error rate of Uyghur is significantly lower than that of English and Chinese in the pre-training using Uyghur alone, and the results obtained after the mixed training of English and Uyghur and Chinese and Uyghur are all better than those obtained after the mixed training of English and Chinese. This phenomenon suggests that Uyghur is more suitable as a source language to transfer knowledge to Kazakh speech recognition tasks than English or Chinese. Since both Uyghur and Kazakh belong to the Turkic language family of the Altaic family, we believe that cross-language knowledge transfer using languages belonging to the same family can achieve more exciting results. More importantly, whenever another language is added to the separate language data, the final word error rate is reduced, suggesting that the model can learn universal phonological features.

When the three languages are mixed for training, the word error rate can achieve the same effect as supervised learning with the E2E-Transformer when only 20 h target language has labeled audio data. When the TTS synthesized Kazakh audio is added for pre-training, the word error rate is further reduced. With only 10 h target language audio data, the identification accuracy rate is similar to that of the E2E-Transformer. This shows that data enhancement methods using speech synthesis can bring huge benefits to speech recognition in the presence of unmatched data.

### 5.4. Contrast with Other Network Layers

In order to prove the effectiveness of the proposed method, we consider replacing the TDNN-F layers with the TDNN layers [43], BiLSTM layers [44], DSFMN layers [45], and TDNN-LSTM layers [46]. Table 7 shows the results after the fusion of different network layers and wav2vec2.0. In these experiments, the audio of Librispeech’s train-clean-100 subset is used as the pre-training data, and the labeled 10 h Kazakh audio is used for fine-tuning and evaluation on the KSC test set. The recognition results of wav2vec-F are optimized while the number of model parameters increases the least.

## 6. Conclusions

This paper proposes wav2vec-F for unsupervised pre-training of speech, using unpaired speech audio and tags for speech recognition, learning the potential speech representations from the waveforms of unlabeled audio data, and applying them to cross-language ASR tasks. On the Librispeech benchmark, WAV2VEC-F outperforms wav2vec 2.0. On the KSC benchmark, WAV2VEC-F outperforms wav2vec 2.0 and previous supervised methods. Meanwhile, the experimental results also prove that multi-language pre-training is more effective than single-language pre-training. It is necessary for low-resource languages to be able to use other accessible, high-resource languages for knowledge transfer. In the pre-training process, better results can be obtained by using a language close to the target language. Compared with supervised training, the method proposed in this paper can make use of audio data unrelated to the target language to carry out speech recognition tasks. Given the same amount of mixed data from other languages, the recognition result is similar to that of supervised learning under the condition that only 10 h of target language data are used for fine-tuning. Furthermore, the recognition effect is optimal when only the target language data are used for pre-training. In our future work, we will continue to explore how training with non-target language data can achieve similar or even better results than training with only target language data.

## Figures and Tables

**Table 1:** Specifications of the LibriSpeech, Primewords Chinese Corpus Set 1, Uyghur (our), and KSC corpus datasets.

Dataset	Durations				Speakers
	Train	Dev	Test	Total	
LibriSpeech	960.9	10.7	10.5	982.1	2484
LibriSpeech (train-clean-100)	100.6	(other) 5.3/(clean) 5.4	(other) 5.1/(clean) 5.4	121.8	251
Uyghur (our)	960.2	10.3	10.1	980.6	3179
Uyghur (train-clean-100)	93.9	5.2	5.2	104.3	198
KSC	318.4	7.1	7.1	332.6	-
Primewords Chinese Corpus Set 1	96.9	1.1	0.9	98.9	296

**Table 2:** Architecture of Factorized-TDNN.

Layer	Layer Type	Context Factor1	Context Factor2	Skip conn. from Layer	Size	Inner Size
1	TDNN-ReLU	t − 2,t + 2			512	
2	F-TDNN-ReLU	t − 2,t	t,t + 2		1024	256
3	F-TDNN-ReLU	t	t		1024	256
4	F-TDNN-ReLU	t − 3,t	t,t + 3		1024	256
5	F-TDNN-ReLU	t	t	3	1024	256
6	F-TDNN-ReLU	t − 3,t	t,t + 3		1024	256
7	F-TDNN-ReLU	t − 3,t	t,t + 3	2,4	1024	256
8	F-TDNN-ReLU	t − 3,t	t,t + 3		1024	256
9	F-TDNN-ReLU	t	t	4,6,8	1024	256
10	Dense-ReLU	t	t		2048	
11	Pooling (mean + stddev)	full-seq			2 × 2048	
13	Dense-ReLU				512	

**Table 3:** Specification of data sets for each language.

Data	Training Utts	Validating Utts	Units
LS 960 h	253,117	28,124	subword
KSC 330 h	132,513	14,723	subword
LS 100 h	25,687	2854	subword
Ma 100 h	45,346	5038	character
Uy 100 h	52,487	5846	subword

**Table 4:** The results (WER) on Librispeech test set when training on the low-resource labeled data setups of 10 min, 1 h, 10 h, and the clean 100 h subset of Librispeech.

Model	Unlabeled Data	Finetune			
		10 min	1 h	10 h	100 h
Baseline	LS 960 h	30.8	27.1	18.4	9.6
wav2vec-F		30.2	26.7	18.2	9.3

**Table 5:** The performance (WER) of different methods on the KSC valid set and test set when pre-training on the train set and fine-tuning on the valid set.

Model	Unlabeled Data	LM	SpeedPerturb	SpecAugment	Valid	Test
DNN-HMM	-	YES	YES	YES	14.9	13.8
E2E-LSTM	-	YES	YES	YES	13.1	11.7
E2E-Transformer	-	YES	YES	YES	10.0	8.7
Baseline	KSC 330 h	YES	No	No	6.4	5.2
wav2vec-F	KSC 330 h	YES	No	No	6.1	5.0

**Table 6:** Results of fine-tuning Kazakh data with different time settings when pre-training with different non-target language data as well as mixed data.

Model	Unlabeled Data	Finetune				
		10 min	1 h	5 h	10 h	20 h
Baseline	LS 100 h	87.7	56.1	37.2	23.6	17.1
	Ma 100 h	77.4	39.3	29.3	20.3	11.5
	Uy 100 h	70.0	35.1	25.2	15.9	10.8
wav2vec-F	LS 100 h	87.5	55.6	34.5	22.1	16.7
	Ma 100 h	77.4	38.0	24.2	15.3	10.5
	Uy 100 h	68.6	34.2	23.5	14.7	10.6
wav2vec-F	LS 100 h + Ma 100 h	61.0	25.7	23.0	15.6	11.4
	LS 100 h + Uy 100 h	58.2	27.9	21.7	13.3	8.9
	Ma 100 h + Uy 100 h	57.4	25.6	20.2	14.5	9.0
	LS 100 h + Ma 100 h + Uy100 h	48.3	25.4	13.0	10.2	8.5
	LS100 h + Ma 100 h + Uy 100 h + TTS 4 h	38.9	19.2	10.8	8.6	6.7

**Table 7:** The results of the fusion of different types of network structures are used, and “+” represents the increased parameter amount.

Converged Network	None (Baseline)	TDNN	BiLSTM	DSFMN	TDNN-LSTM	TDNN-F
WER (%)	23.6	24.3	23.2	22.4	22.9	22.1
Params (M)	-	+19	+41	+27	+40	+20

## Data Availability

Not applicable.
